# Alterations in the gut microbiome and metabolome profiles of septic rats treated with aminophylline

**DOI:** 10.1186/s12967-022-03280-3

**Published:** 2022-02-03

**Authors:** Yuanzhe Li, Huayan Zhao, Guiying Sun, Yongtao Duan, Yanjun Guo, Lina Xie, Xianfei Ding

**Affiliations:** 1 Department of Pediatrics, Children’s Affiliated Hospital of Zhengzhou University, Zhengzhou, China; 2 Department of Critical Care Medicine, The First Affiliated Hospital of Zhengzhou University, Zhengzhou, China; 3 Epidemiology and Statistics, College of Public Health, Zhengzhou University, Zhengzhou, 450001 China; 4 General Intensive Care Unit, The First Affiliated Hospital of Zhengzhou University, Zhengzhou, China

## Abstract

The treatment of sepsis remains a major challenge worldwide. Aminophylline has been shown to have anti-inflammatory effects; however, the role of aminophylline in sepsis, a disease characterized by immune dysregulation, is unknown. In this study, we combined microbiome sequencing and metabolomic assays to investigate the effect of aminophylline administration on the intestinal flora and metabolites in septic rats. Sixty SD rats were randomly divided into three groups: a sham-operated (SC) group, a sepsis model (CLP) group and a CLP + aminophylline treatment (Amino) group. The intestinal flora and metabolic profile of rats in the CLP group were significantly different than those of the SC group, while aminophylline administration resulted in a return to a state similar to healthy rats. Differential abundance analysis showed that aminophylline significantly back-regulated the abundance of *Firmicutes*, *unidentified_Bacteria*, *Proteobacteria*, *Lactobacillus*, *Escherichia-Shigella* and other dominant bacteria (*P* < 0.05) and altered chenodeoxycholic acid, isolithocholic acid and a total of 26 metabolites (variable importance in the projection (VIP) > 1, *P* < 0.05). In addition, we found that there were significant correlations between differential metabolites and bacterial genera of the Amino and CLP groups. For example, *Escherichia-Shigella* was associated with 12 metabolites, and *Lactobacillus* was associated with two metabolites (*P* < 0.05), suggesting that differences in the metabolic profiles caused by aminophylline were partly dependent on its influence on the gutmicrobiome. In conclusion, this study identified a novel protective mechanism whereby aminophylline could regulate disordered intestinal flora and metabolites in septic rats.

**Supplementary Information:**

The online version contains supplementary material available at 10.1186/s12967-022-03280-3.

## Introduction

Sepsis is a dysregulated immune response to infection that leads to organ dysfunction, and sepsis affects more than 30 million people worldwide each year [1, 2]. Despite intensive research on the pathogenesis and treatment of sepsis in recent years, the morbidity and mortality of sepsis remain high in clinical practice [3–5]. The treatment of sepsis remains a major challenge worldwide.

Inflammatory imbalance is one of the most critical bases for the pathogenesis of sepsis. The initial acute response of the host to infection usually elicits a series of proinflammatory cytokines that achieve rapid control of minor and localized infections. However, when the response exceeds a certain threshold, it causes a cytokine storm, leading to multiorgan damage or even life-threatening situations [3, 6, 7]. Therefore, downregulating the inflammatory immune response early in sepsis is thought to potentially improve the patient’s prognosis. Aminophylline, a nonselective adenosine receptor antagonist and phosphodiesterase inhibitor [8, 9], has been shown to have anti-inflammatory effects [10–13]. For example, aminophylline inhibits the hydrolysis of cyclic adenosine monophosphate (cAMP) and thus increases intracellular cAMP concentration [9, 14], decreases the expression of pro-inflammatory cytokines and nuclear factor (NF-kB) [15, 16], and reduces endothelial permeability. In addition, aminophylline pre-treatment reduces the release of troponin I and the activation of neutrophils in the myocardium of patients with cardiac arrest [17]. However, the role of aminophylline in sepsis remains unclear.

In recent years, the understanding of the role of the gut microbiome and metabolome in sepsis has increased; currently the gut is thought to be closely associated with sepsis pathogenesis and outcome [18]. In this study, we hypothesized that aminophylline could at least partially modulate the intestinal flora and faecal metabolites and thus affect the outcome of sepsis in a rat model.

## Methods

### Animals and experimental procedure

The experimental animals were Sprague–Dawley rats (6–8 weeks old, all males) from Beijing Viton Lever Experimental Co., Ltd. The rats were acclimatized to the environment and provided with sufficient food and water before being randomly divided into 3 groups (n = 20/group): the sham operation (SC) group, the sepsis model (CLP) group, and the sepsis + aminophylline administration (Amino) group. Sepsis was induced by cecal ligation and puncture method, and which is based on our previous research [19]. Briefly, the rat abdomen was shaved and thoroughly cleaned with complex iodine following intraperitoneal injection of 10% chloral hydrate (350 mg/kg), and surgeries were conducted on a sterilized board. Approximately 2.0 incisions were made at the midline of the abdomen. The cecum was squeezed until a small amount of fecal material was excreted and then returned to the peritoneal cavity. The abdominal cavity was then sutured with aseptic 5 + 0 surgical sutures, and the skin was closed using a septic 3 ± 0 surgical sutures. After the operation, all rats were immediately placed in a warm environment and subcutaneously injected with preheated physiological saline (50 mL/kg) for fluid resuscitation. Rats in the Amino group were additionally injected with 50 mg/kg aminophylline intraperitoneally at 1 h after surgery, which were based on our pre-experimental survival results. Rats in the SC group underwent the same surgical operation as the CLP and Amino groups, except that the caecum was not ligated or punctured. The sepsis model for rats in the CLP and Amino groups was constructed with reference to previous literature [20]. The survival status of the rats in each group was recorded at 24 h postoperatively, after which the faeces were collected and stored at − 80 °C for freezing.

### Genomic DNA extraction and 16S rRNA sequencing

Faecal DNA was extracted by the CTAB method; the concentration was detected by agarose gel electrophoresis and diluted to 1 ng/µl with sterile water. Subsequently, PCR amplification of the V3-V4 region of the 16S rRNA gene was performed using the diluted DNA as a template. Finally, 16S rRNA sequencing was performed using NovaSeq6000.

### Sequencing data analysis

Raw tags were obtained by stitching together the reads of each sample using FLASH software [21] and filtered using QIIME (V1.9.1) to obtain clean tags [22, 23]. Afterwards, the final effective tags were obtained by (https://github.com/torognes/vsearch/) matching with the species annotation database and removing chimeric sequences. All effective tags were clustered using Uparse (v7.0.1001), and sequences with a similarity threshold above 97% were assigned to operational taxonomic units (OTUs) by default. Species annotation was performed with the SSUrRNA database [24] using the Mothur method (set threshold of 0.8–1). The alpha and beta diversity indices were calculated using QIIME software, and LEfSe analysis was performed using LEfSe software with the default setting of a screening value of 4 for the LDA score. Other plots, such as rarefaction curves, PCoA plots, and plots of significant differences in species between groups, were created in R (v2.15.3).

### Non-targeted metabolomics assays

Twenty-five milligrams of stool was placed into an Eppendorf (EP) tube, and 500 µl of extraction solution (methanol: acetonitrile: water = 2:2:1 (V/V), containing isotopically labelled internal standard mixture) was added. The solution was mixed well and allowed to stand at − 40 °C for 1 h before centrifugation for 15 min (4 °C, 12,000 rpm). The supernatant was collected in an injection vial for the assay.

The target compounds were chromatographically separated on a Waters ACQUITY UPLC BEH Amide (2.1 mm × 100 mm, 1.7 μm) liquid chromatographic column using a Vanquish (Thermo Fisher Scientific) ultra-performance liquid chromatograph. The A phase of the liquid chromatography was aqueous, containing 25 mmol/L ammonium acetate and 25 mmol/L ammonia, and the B phase was acetonitrile. The sample tray temperature was 4 °C, with an injection volume of 2 μL. Finally, primary and secondary mass spectrometry data acquisition was performed by a Q Exactive HF-X (Thermo Fisher Scientific) mass spectrometer.

### Metabolomics data analysis

The raw data were first converted to mzXML format by Proteo Wizard software, and peak identification, peak extraction, peak alignment and integration were performed using an in-house R package (kernel XCMS) [25] before data were matched with the BiotreeDB (V2.1) self-built secondary mass spectrometry database for substance annotation (cut-off value = 0.3). After data management [26] of the raw data, univariate and multivariate statistical analyses of the qualitative and quantitative results of the metabolic groups were performed. The permutation test was used to evaluate the robustness of the OPLS-DA model, and differential metabolites were defined as metabolites with a variable importance in the projection (VIP) > 1 and *P* < 0.05. KEGG annotation and pathway enrichment analysis of differential metabolites were conducted in combination with the Kyoto Encyclopedia of Genes and Genomes (KEGG) Pathway database (http://www.kegg.jp/kegg/pathway.html). R software was used to visualize the differential metabolites and pathways.

### Statistical analysis

T tests, Wilcox rank sum tests and Tukey tests were used to analyse whether the species differences between groups were significant. MetaStat analysis was performed at each taxonomic level to obtain p values for the permutation test between groups; p values were corrected using the Benjamini and Hochberg false discovery rate [27] method to obtain q-values. Species with significant differences were screened according to the q-values. Correlation analysis was performed using Pearson correlations and Spearman correlations.

## Results

### Aminophylline increased survival in septic rats

To determine whether aminophylline has a protective effect on septic rats, we first examined mortality rates across the three groups (Additional file 3: Table S1). The results showed a significant increase in 24-h mortality (55%) in CLP-induced septic rats compared to the sham-operated group (*P* = 0.002); the mortality rate decreased to 30% in the Amino group, although the difference between the CLP and Amino groups was not statistically significant (*P* = 0.110).

### Gut flora diversity analysis of rats

**Fig. 1:**
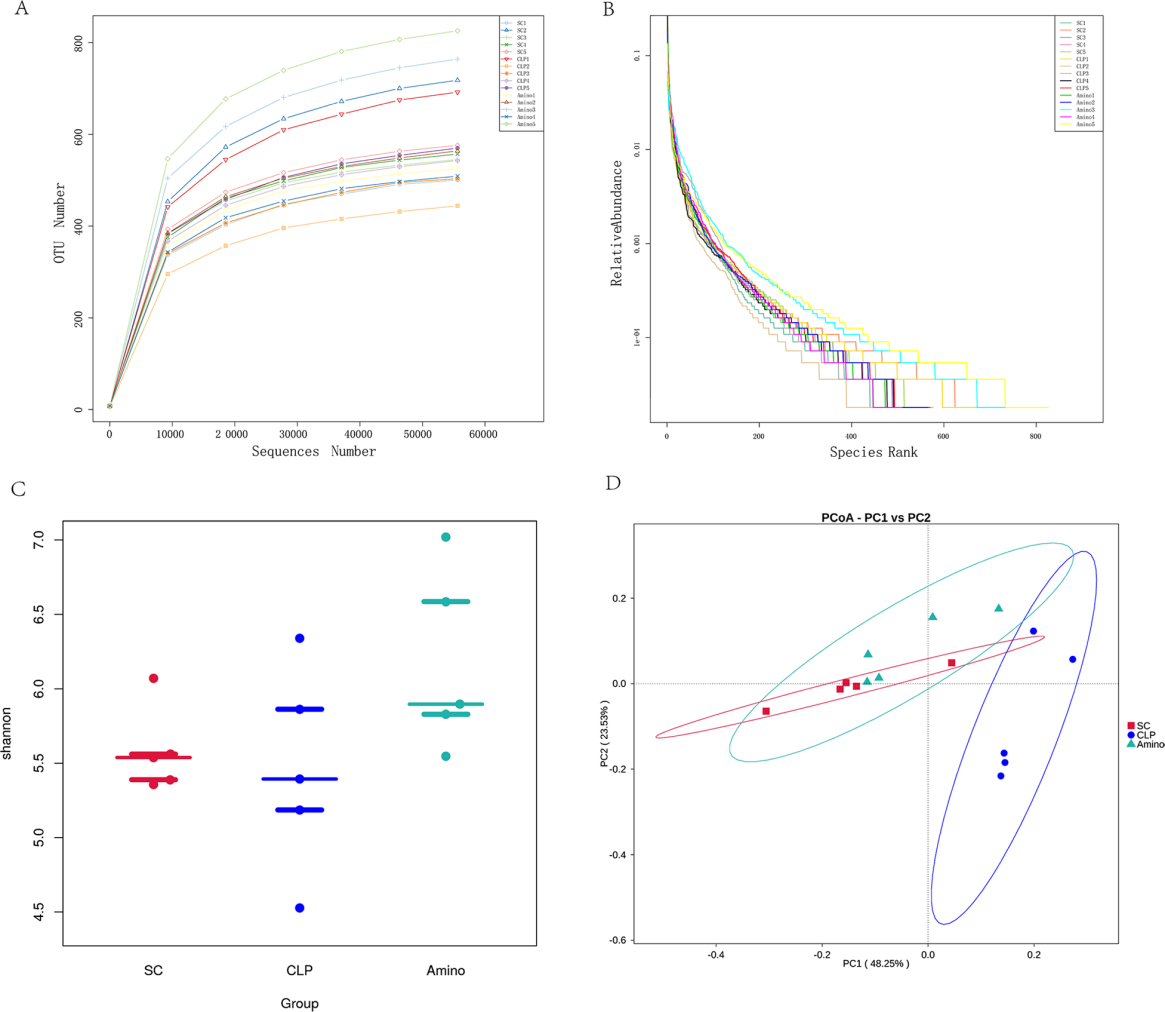
Gut microbiome diversity analysis. **A** Rarefaction curve and **B** rank abundance curve showing the plausibility of the sequencing data and indirectly reflecting species richness in the samples. **C** The Shannon index, showing the alpha diversity in each group. **D** PCoA, based on weighted uniFrac distance, showing the beta diversity of each group

To verify whether aminophylline exerts beneficial effects at least partially through the gut microbiome, we performed 16S rRNA sequencing of the rat faeces. In addition, the sequencing depth was evaluated. Figure 1A shows that the curve flattened as the number of sequences increased, indicating that the results of this sequencing were reasonably plausible. We also generated a rank abundance curve (Fig. 1B), which further confirmed the above point. Then, to compare the overall structural characteristics of the intestinal flora of the three groups, we compared the Shannon index of each group (Fig. 1C) and performed PCoA (Fig. 1D). The CLP group was distant from the SC group, but the Amino and SC groups were clustered together, indicating that the intestinal flora of rats was significantly altered by sepsis, but the flora structure became similar to that of healthy rats after aminophylline administration.

### Aminophylline modulated the abundance of bacterial flora in septic rats

**Fig. 2:**
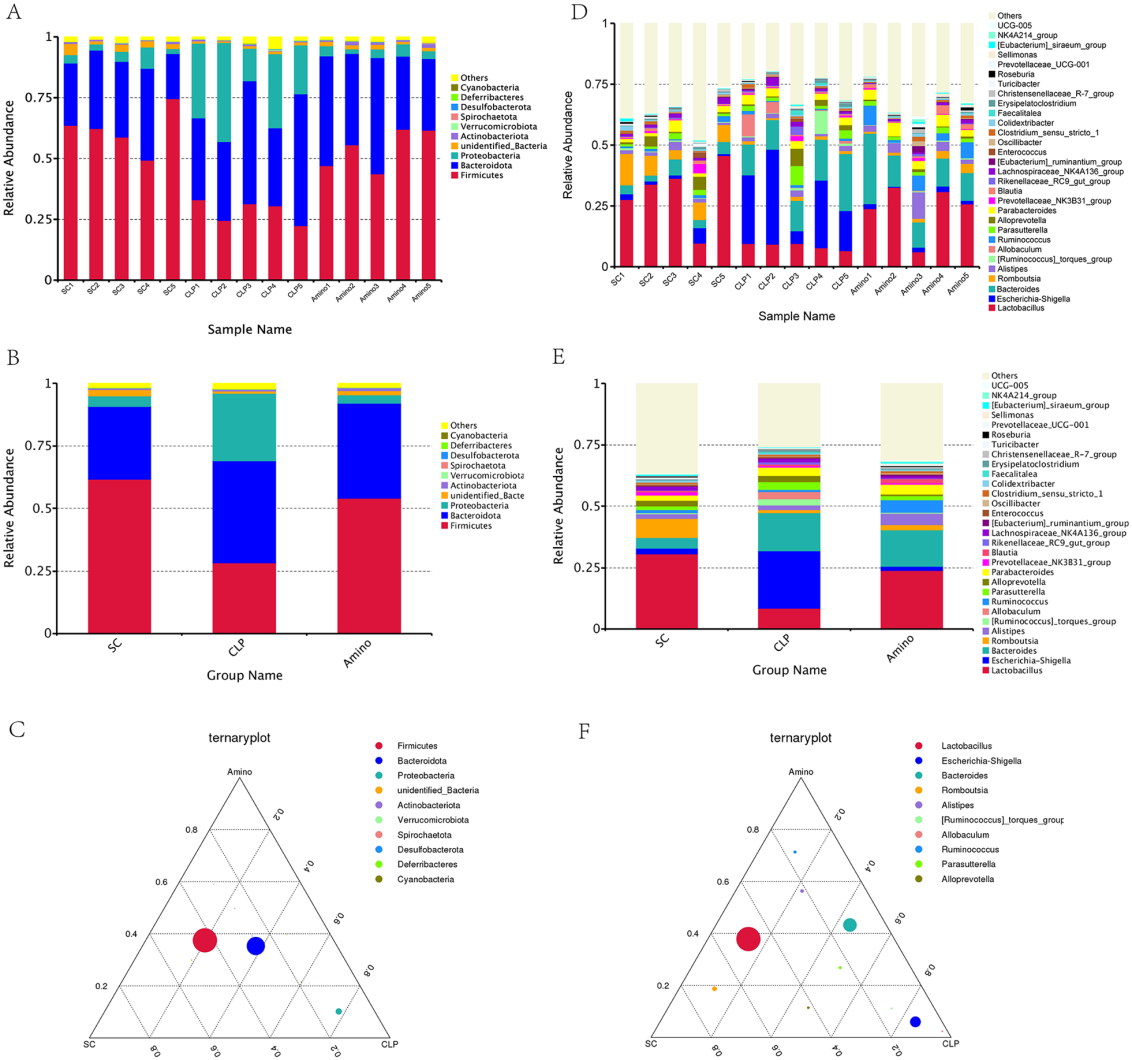
Relative abundance analysis of dominant bacteria at the phylum (**A**–**C**) and genus (**D**–**F**) levels

**Fig. 3:**
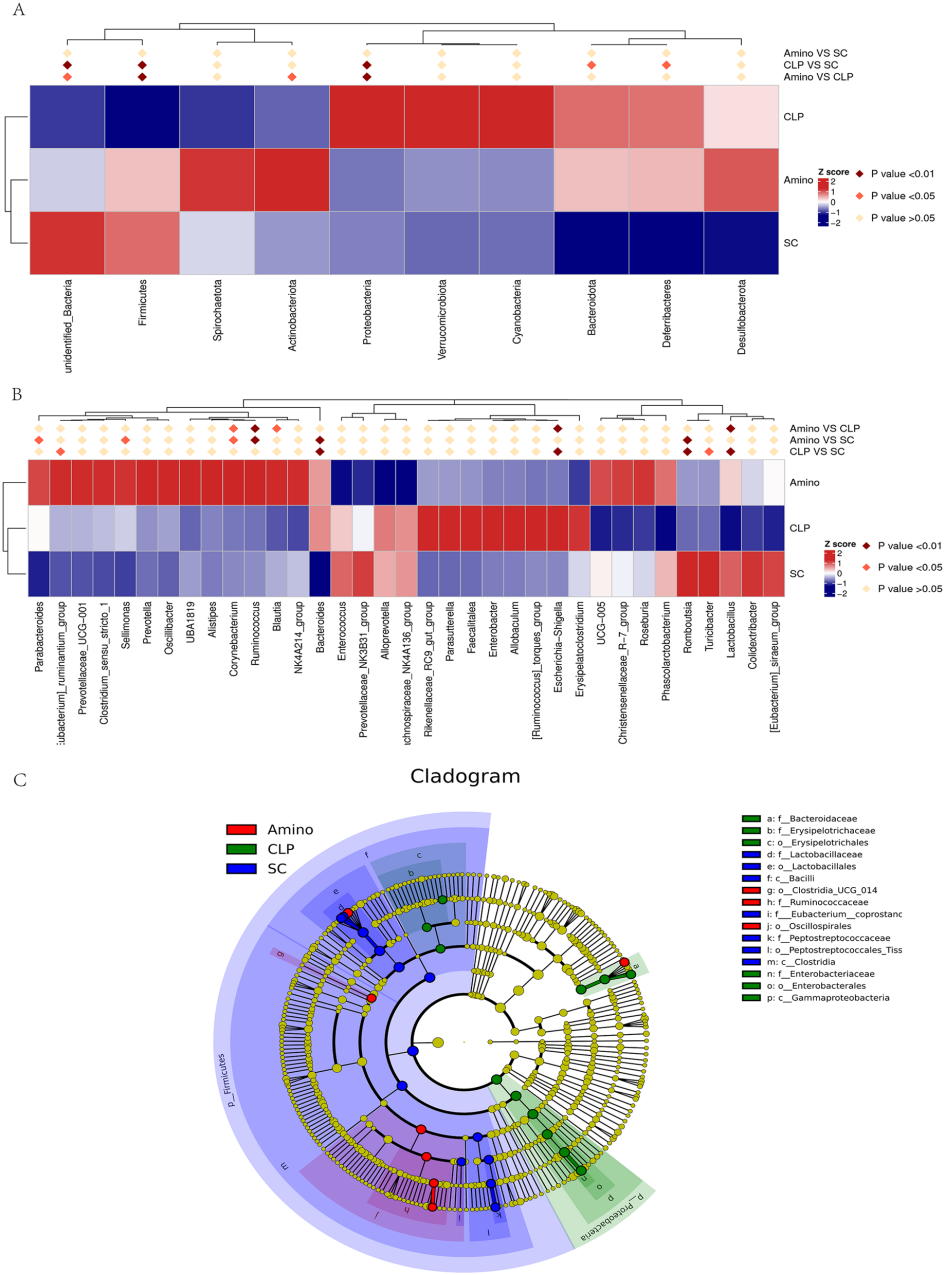
Differential bacteria identification of each group. **A** Meta Statanalysis of the 10most abundant bacteria at the phylum level. **B** Meta Statanalysis of the 30 most abundant bacteria at the genus level. **C** Cladogram-based linear discriminant analysis (LDA) integrated with effect size (LEfSe)

We then focused on bacterial abundance at each taxonomic level, particularly the dominant groups at the phylum and genus levels. Figure 2A–C shows that the Amino and SC groups had similar bacteria as the top ten most abundant phyla, but the CLP group was more variable. For example, *Firmicutes* and *unidentified_Bacteria* decreased in abundance in the CLP group, but *Proteobacteria* and *Spirochaeta* increased in abundance; interestingly, these changes were restored after aminophylline administration. We then analysed the top30 most abundant bacteria at the genus level (Fig. 2B–D). Similar to the distribution characteristics at the phylum level, the abundance of bacteria such as *Lactobacillus and Romboutsia* decreased in the CLP group compared to the SC group and increased in the Amino group; *Escherichia-Shigella* and *Allobaculum* increased in abundance in the CLP group but decreased to levels similar to those in healthy rats after aminophylline administration. MetaStat analysis was then used to further screen for statistically significant differences in bacteria, and the results showed that the above differences between the above *Firmicutes*, *unidentified_Bacteria*, *Proteobacteria* phylum and *lactobacillus*, *Escherichia-Shigella* genera were statistically significant, which empirically demonstrates the beneficial effect of aminophylline on the abnormal intestinal flora of septic rats (Fig. 3A, B). In addition, we performed LEfSe analysis and plotted cladogram (Fig. 3C), which allowed us to determine that CLP group-specific bacteria included *Enterobacteriaceae* and *Bacteroidaceae*; these CLP group-specific bacteria were significantly reduced or even disappeared after aminophylline administration.

### Aminophylline alters the faecal metabolic profile in septic rats

**Fig. 4:**
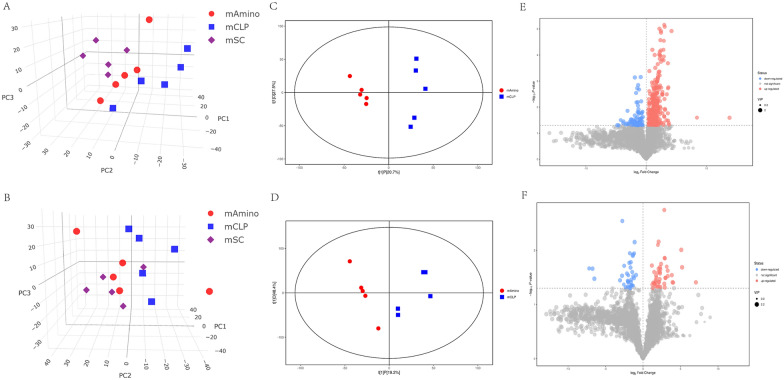
Overall metabolic characteristics in each group. **A**, **B** PCA scores of three groups. **C**, **D** OPLS-DA scores of CLP and Amino group. **C** Volcano plot of metabolite distribution between the Amino andCLPgroups. **A**, **C**, **E** Negative ion mode. **B**, **D**, **F** Positive ion mode

In general, bacteria usually affect certain specific metabolites and thus the immune status of the host [28, 29]. Therefore, we further analysed the faecal metabolome to look for alterations in septic rats after treatment with aminophylline. First, we determined the total ion chromatograms of the groups (Additional file 1: Fig. S1), and we found that the peak pattern of the Amino group was more similar to that of healthy rats than the peak pattern of the CLP group. We then created a 3D PCA score plot for the three metabolite groups to visualize overall changes in metabolites (Fig. 4A, B). The results showed significant differences between the CLP and SC groups both in positive and negative ion mode, while the Amino group had a significant tendency to retrace, which suggested that aminophylline modulated the metabolites of septic rats to approach those of healthy rats. We also performed OPLS-DA, and similar to the PCA results, both the CLP and SC groups differed significantly under positive and negative ions (Additional file 2: Fig. S2A, B), indicating that the metabolic profile of rats was significantly altered in sepsis. The metabolites after aminophylline administration were also significantly different from those of the CLP group (Fig. 4C, D), suggesting that aminophylline altered the abnormal metabolic profile of septic rats. In addition, we used a volcano plot containing all substances measured in this experiment to visualize the overall distribution of metabolite differences between groups under positive and negative ions (Fig. 4E, F; Additional file 2: Fig. S2C, D). In conclusion, these results suggest that aminophylline alters the metabolites in septic rats to levels similar to those in healthy rats.

### Differential metabolite and pathway analysis in rats

**Fig. 5:**
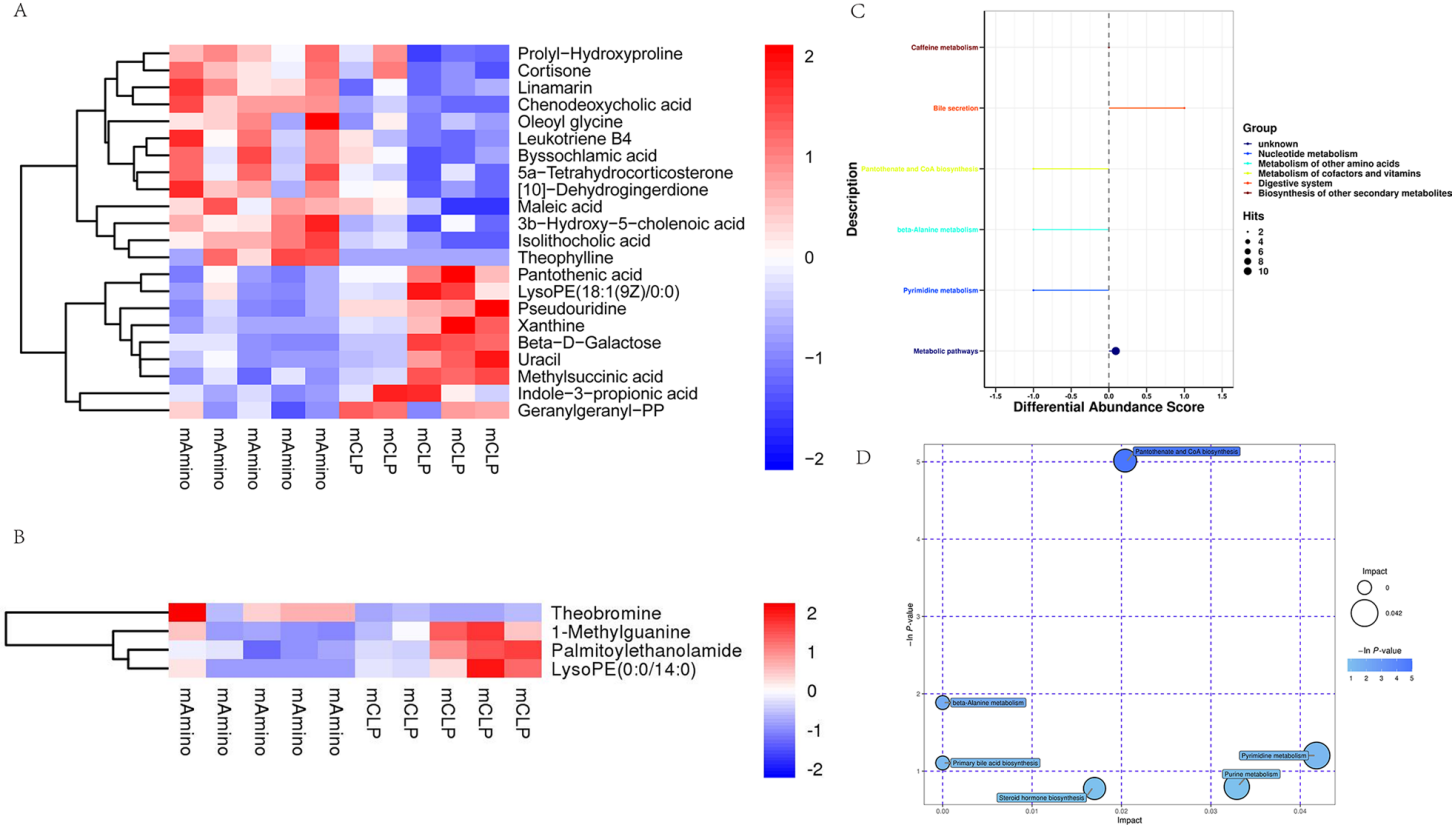
Differential metabolite and pathway analysis. **A** Negative ion mode. **B** Positive ion mode. **C** Differential abundance analysis scores. **D** Pathway analysis

For further screening of aminophylline-related metabolites, the value of VIP of the first principal component in OPLS-DA was obtained, which summarized the contribution of each variable to the model. Metabolites with VIP > 1 and *P* < 0.05 (Student’s t test) were considered to be significantly changed metabolites. Finally, a total of 26 differential metabolites were screened between the Amino and CLP groups (Additional file 4: Table S2). Figure 5A and B show the content of differential metabolites in each sample under positive and negative ions. Maleic acid, isolithocholicacid, oleoyl glycine, leukotriene B4, by ssochlamicacid, 5a-tetrahydrocorticosterone, [10]-dehydrogingerdione, prolyl-hydroxyproline, cortisone, linamarin, 3b-hydroxy-5-cholenoic acid, chenodeoxycholic acid, andtheophyllinewere increased by aminophylline treatment. Pantothenic acid, lysoPE (18:1(9Z)/0:0), pseudouridine, xanthine, beta-D-galactose, uracil, methylsuccinicacid, indole-3-propionic acid, geranylgeranyl-PP, 1-methylguanine, palmitoylethanolamide, and lysoPE (0:0/14:0) were decreased.

In addition, commercial databases, including KEGG (http://www.genome.jp/kegg/) and MetaboAnalyst (http://www.metaboanalyst.ca/), were used for pathway enrichment analysis. The results showed that aminophylline mainly affected metabolic pathways, pyrimidine metabolism, beta-alanine metabolism, pantothenate and CoA biosynthesis, as well as other pathways (Fig. 5C, D).

### Associations between differential genera and metabolites

**Fig. 6:**
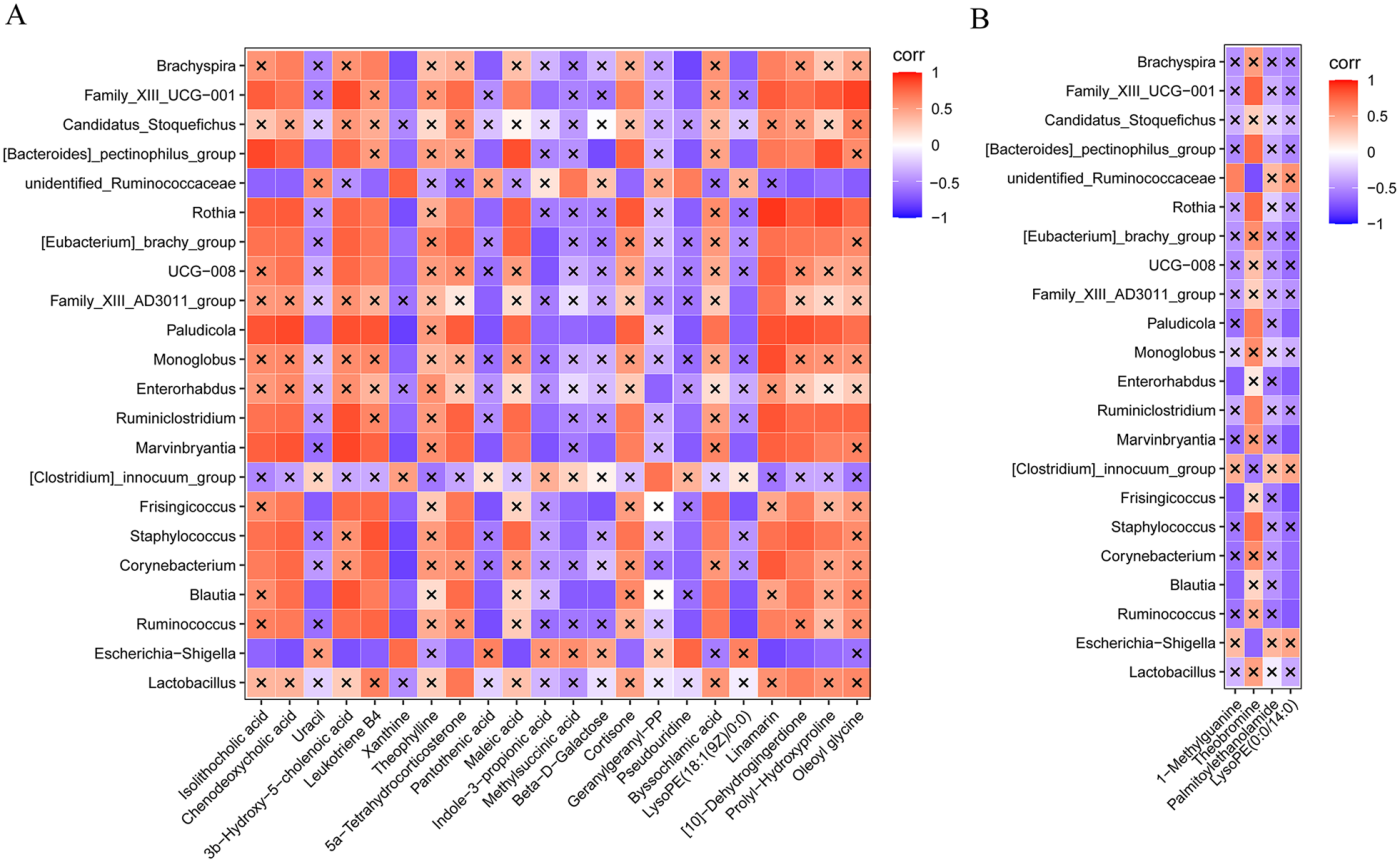
Correlation analysis between differential genera and metabolites. **A** Negative ion mode. **B** Positive ion mode. ×: *P* value > 0.05

To investigate whether the altered metabolite abundance induced by aminophylline treatment was correlated with intestinal flora, we performed a Spearman correlation between differential flora and differential metabolites at the genus level in the Amino and CLP groups. We found a significant correlation between 22 differential genera and 26differential metabolites (Fig. 6). Some bacteria enriched in amino acids, such as *Lactobacillus*, were found to be associated with 5a-tetrahydrocorticosterone and [10]-dehydrogingerdione, while *Escherichia-Shigella* decreased in abundance after aminophylline treatment and was associated with isolithocholic acid, chenodeoxycholic acid, 3b-hydroxy-5-cholenoic acid, leukotriene B4, xanthine, 5a-tetrahydrocorticosterone, maleic acid, linamarin, [10]-dehydrogingerdione, prolyl-hydroxyproline, pseudouridine, cortisone and pseudouridine.

## Discussion

Many studies have demonstrated the anti-inflammatory effects of aminophylline. First, as a well-known phosphodiesterase inhibitor, aminophylline significantly increases cAMP levels [14], which could regulate inflammatory mediators such as caspase-11 and cAMP response element binding, as well as nuclear factor kappa B, and thus avoid excessive inflammatory responses in sepsis [30, 31]. Aminophylline also inhibits the activation of some inflammatory cells, such as neutrophils, and the expression of some cytokines, such as interleukin-1β [13, 14]. In addition, aminophylline has been found to reduce endothelial cell permeability and promote vascular endothelial integrity [32], which plays a crucial role in sepsis as the pathological mechanism of organ damage [33]. Therefore, it can be assumed that aminophylline is a promising candidate for the treatment of sepsis.

Intestinal flora has been shown to be closely associated with the development of sepsis. Singer JR et al. showed that the prevention of microbial dysbiosis in mice mitigated the spread of pathogens in the intestine and protected against late-onset sepsis [34]. Targeting the intestinal flora holds promise as a new therapeutic target for sepsis [35].Our study showed that aminophylline modulated the abundance of bacteria such as *Firmicutes*, *Proteobacteria*, *Escherichia_Shigella*, and *Lactobacillus*, returning them to levels similar to those of healthy rats (*P* < 0.05), which suggests that these bacteria may mediate the beneficial effects of aminophylline. Of these, *Lactobacillus*, taxonomically belonging to the *Firmicutes* phylum, showed a significant increase in abundance after aminophylline treatment. *Lactobacillus*is considered a probiotic with powerful health-promoting effects in different environments [36]. Previous studies have shown that *Lactobacillus* can maintain microbial homeostasis and epithelial barrier integrity in the host environment, inhibit pathogenic bacterial invasion and colonization, and modulate the host immune response through a variety of mechanisms [37–39]. *Escherichia_Shigella*, taxonomically belonging to *Proteobacteria*, is one of the most common intestinal pathogenic bacteria characterized by invasion and destruction of the human colonic epithelium. It transfers virulence proteins directly from bacteria to the cytoplasm of host cells through the three secretion system (T3SS), thus subverting the function of epithelial cells and manipulating immune cells to cause their dysfunction and disrupt the immune homeostasis in the host [40, 41]. As seen above, aminophylline could modulate the gut microbiota by increasing beneficial bacteria and decreasing pathogenic bacteria in septic rats, which has not been reported in previous mechanistic studies of aminophylline.

Considering that multiple bacteria in the gut can regulate metabolic reactions, such as the production of bile acids and fatty acids, which are essential for the health of the host [42], we performed nontargeted metabolic profiling and found that aminophylline significantly altered the levels of 22 metabolites in septic rats (*P* < 0.05, Fig. 5). Among these metabolites, bile acids (isolithocholicacid, 3b-hydroxy-5-cholenoic acid and chenodeoxycholic acid) were the most abundant differential metabolites. It is well known that bile acids play a key role in regulating hepatic metabolic pathways [43], which in turn are critical for regulating immune defence in sepsis due to mechanisms such as bacterial clearance and metabolic adaptation to inflammation [44]. Previous studies have shown that obeticholic acid, a derivative of chenodeoxycholic acid, can improve bile acid homeostasis; inhibit the expression of TNF-α, IL-6, and IL-1β; and alleviate sepsis-related liver injury, which suggests that bile acids have a protective effect against sepsis [45]. It is also worth mentioning that one of the differential metabolites, theophylline, was almost absent in the CLP group and significantly increased in the Amino group, which indicates that aminophylline administration during the experiment was effective. In addition, the results of the association analysis showed that the differential bacteria were not correlated with theophylline, while the other metabolites were more or less correlated with the bacteria; therefore, it can be assumed that the changes in the ophylline content were due to drug administration, while the changes in other metabolites were at least indirectly caused by changes in the bacterial flora.

This study also had several limitations. The sample size of each group of rats was small, and we did not evaluate the possible side effects of aminophylline administration in septic rats. A wider sample of animal models and more detailed molecular biology experiments are needed to explore clinical translations of aminophylline for sepsis treatment.

## Conclusion

This study found that aminophylline exerted beneficial effects on sepsis by modulating the abnormal intestinal bacterial structure and affecting its metabolites. This was the first study to investigate the effect of aminophylline on the gut microbiome of septic rats and may provide new insights into the therapeutic use of aminophylline in sepsis.

## Supplementary Information

**Additional file 1: Figure S1**. Total ion chromatograms of three groups. (A-C) Negative ion mode. (D-F) Positive ion mode.

**Additional file 2: Figure S2.** OPLS-DA scores and volcano plot of the CLP and SC groups. (A, C) Negative ion mode. (B, D) Positive ion mode.

**Additional file 3: Table S1.** 24-h mortality of animals in each group.

**Additional file 4: Table S2.** Major differential metabolites between the Amino and CLP groups.

## Data Availability

All datasets generated for this study are included in the article/additional file.
