# Supplementary figures and images for: Alterations in the gut microbiome and metabolome profiles of septic rats treated with aminophylline
Source: J Transl Med. 2022 Feb 3;20:69. doi: 10.1186/s12967-022-03280-3 (PMC8812188; doi:10.1186/s12967-022-03280-3)

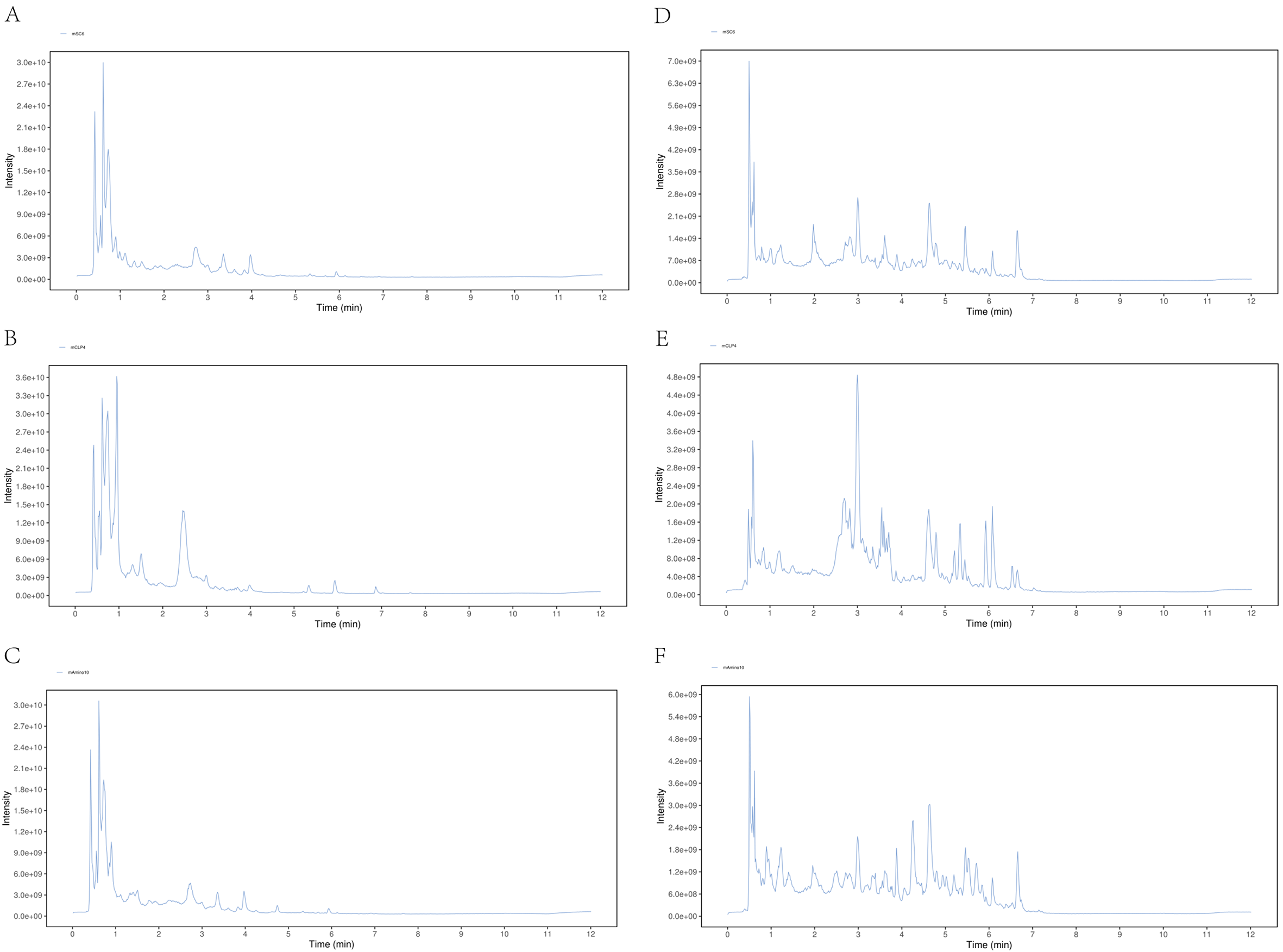

Supplement: Supplementary file 1 — Additional file 1: Figure S1. Total ion chromatograms of three groups. (A-C) Negative ion mode. (D-F) Positive ion mode. [file 12967_2022_3280_MOESM1_ESM.tif]

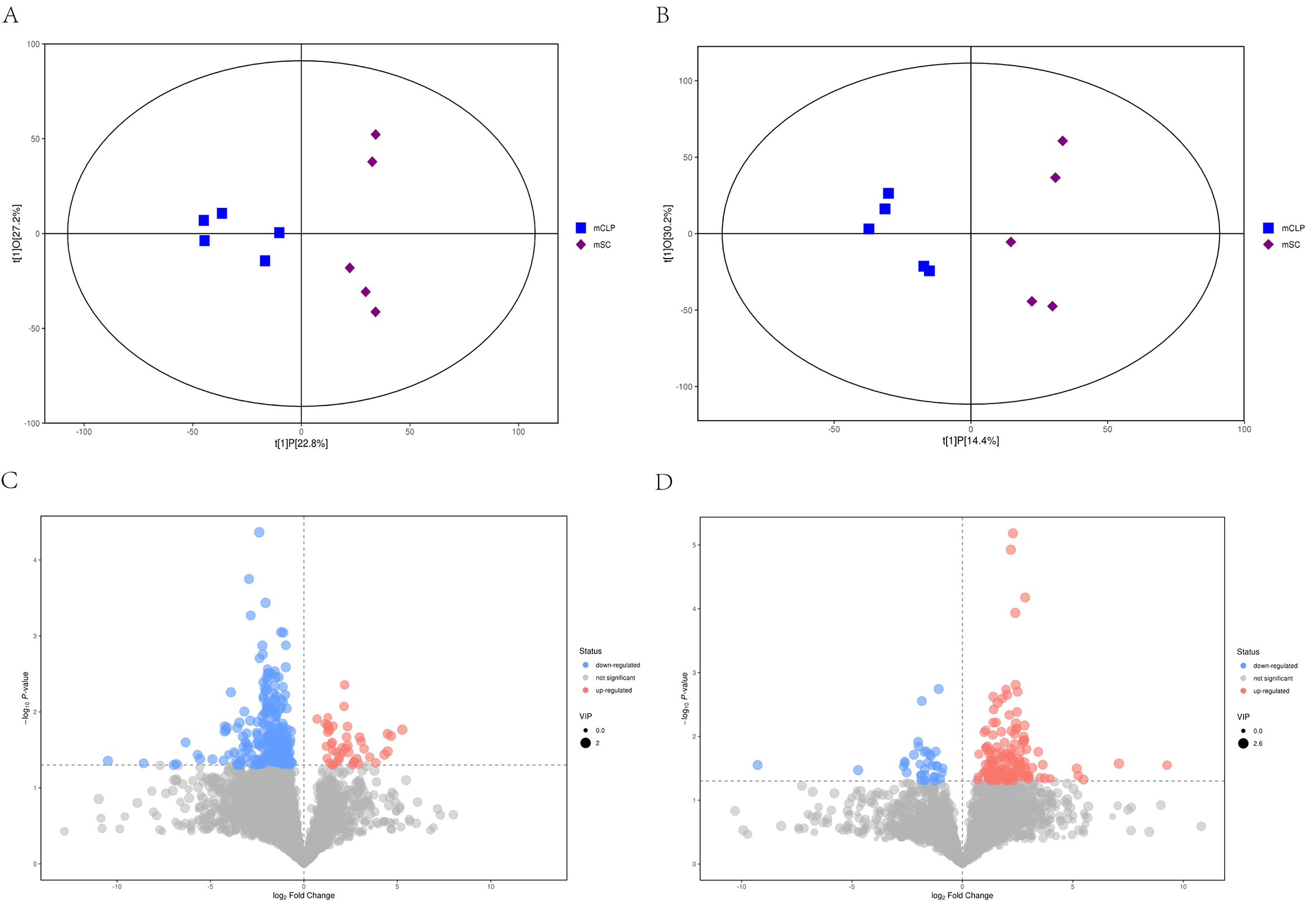

Supplement: Supplementary file 2 — Additional file 2: Figure S2. OPLS-DA scores and volcano plot of the CLP and SC groups. (A, C) Negative ion mode. (B, D) Positive ion mode. [file 12967_2022_3280_MOESM2_ESM.tif]
