# Supplementary material for: Alterations in the gut microbiome and metabolome profiles of septic rats treated with aminophylline
Source: J Transl Med. 2022 Feb 3;20:69. doi: 10.1186/s12967-022-03280-3 (PMC8812188; doi:10.1186/s12967-022-03280-3)
Supplement: Supplementary file 3 — Additional file 3: Table S1. 24-h mortality of animals in each group. [file 12967_2022_3280_MOESM3_ESM.docx]

**Additional file 3: Table S1.** 24-h mortality of animals in each group

| Group | Fatality rate (deaths/total) | *P* value |
| --- | --- | --- |
| SC | 0% (0/20) | 0.001^a^ |
| CLP | 50% (10/20) | 0.197^b^ |
| Amino | 30% (6/20) |  |

^a^Compared with CLP group.

^b^Compared with AMINO group.
