# Supplementary material for: Alterations in the gut microbiome and metabolome profiles of septic rats treated with aminophylline
Source: J Transl Med. 2022 Feb 3;20:69. doi: 10.1186/s12967-022-03280-3 (PMC8812188; doi:10.1186/s12967-022-03280-3)
Supplement: Supplementary file 4 — Additional file 4: Table S2. Major differential metabolites between the Amino and CLP groups. [file 12967_2022_3280_MOESM4_ESM.docx]

**Additional file 4: Table S2.**Major differential metabolites between Amino and CLP groups

| **MS2 name** | **Mode** | **Rt** | **mz** | **Mean（Amino）** | **Mean**  **（CLP）** | **VIP** | ***P* value** | **FC(Amino/CLP)** | **Super class** |
| --- | --- | --- | --- | --- | --- | --- | --- | --- | --- |
| Maleic acid | NEG | 50.48 | 115.003 | 0.359 | 0.188 | 1.466 | 0.040 | 1.91 | Organic acids and derivatives |
| Isolithocholic acid | NEG | 66.93 | 375.291 | 91.066 | 29.883 | 1.852 | 0.001 | 3.047 | Lipids and lipid-like molecules |
| Theophylline | NEG | 56.07 | 179.057 | 57.153 | 0.168 | 1.494 | 0.025 | 339.288 | Organoheterocyclic compounds |
| Oleoyl glycine | NEG | 104.39 | 338.270 | 0.981 | 0.332 | 1.368 | 0.041 | 2.951 | Organic acids and derivatives |
| Leukotriene B4 | NEG | 60.40 | 335.223 | 1.892 | 0.632 | 1.620 | 0.014 | 2.994 | Lipids and lipid-like molecules |
| Byssochlamic acid | NEG | 89.34 | 331.119 | 3.390 | 1.576 | 1.328 | 0.045 | 2.151 | Organoheterocyclic compounds |
| 5a-Tetrahydrocorticosterone | NEG | 59.62 | 349.238 | 5.084 | 2.234 | 1.358 | 0.019 | 2.275 | Lipids and lipid-like molecules |
| [10]-Dehydrogingerdione | NEG | 78.31 | 345.211 | 0.625 | 0.229 | 1.363 | 0.024 | 2.726 | Phenylpropanoids and polyketides |
| Prolyl-Hydroxyproline | NEG | 322.03 | 227.103 | 0.652 | 0.297 | 1.455 | 0.030 | 2.197 | Organic acids and derivatives |
| Cortisone | NEG | 153.22 | 359.189 | 24.945 | 12.706 | 1.475 | 0.046 | 1.963 | Lipids and lipid-like molecules |
| Linamarin | NEG | 132.39 | 246.099 | 1.048 | 0.254 | 1.633 | 0.002 | 4.132 | Organic oxygen compounds |
| 3b-Hydroxy-5-cholenoic acid | NEG | 62.61 | 373.275 | 2.569 | 1.178 | 1.674 | 0.005 | 2.180 | Lipids and lipid-like molecules |
| Chenodeoxycholic acid | NEG | 164.43 | 391.285 | 271.155 | 57.143 | 1.874 | 0.001 | 4.745 | Lipids and lipid-like molecules |
| Pantothenic acid | NEG | 291.86 | 218.103 | 0.743 | 1.819 | 1.848 | 0.012 | 0.408 | Organooxygen compounds |
| LysoPE(18:1(9Z)/0:0) | NEG | 220.39 | 478.295 | 1.690 | 4.292 | 1.693 | 0.039 | 0.394 | Lipids and lipid-like molecules |
| Pseudouridine | NEG | 259.74 | 243.062 | 0.084 | 0.234 | 1.973 | 0.001 | 0.099 | Nucleosides, nucleotides, and analogues |
| Xanthine | NEG | 231.15 | 151.025 | 2.467 | 8.895 | 1.900 | 0.038 | 0.277 | Organoheterocyclic compounds |
| Beta-D-Galactose | NEG | 312.22 | 179.055 | 0.228 | 1.035 | 1.682 | 0.030 | 0.221 | Organic oxygen compounds |
| Uracil | NEG | 79.14 | 111.019 | 60.491 | 112.043 | 1.580 | 0.042 | 0.540 | Organoheterocyclic compounds |
| Methylsuccinic acid | NEG | 383.11 | 131.034 | 0.182 | 0.312 | 1.561 | 0.018 | 0.582 | Lipids and lipid-like molecules |
| Indole-3-propionic acid | NEG | 97.54 | 188.071 | 0.646 | 2.185 | 1.647 | 0.043 | 0.296 | Organoheterocyclic compounds |
| Geranylgeranyl-PP | NEG | 53.96 | 449.185 | 0.573 | 0.898 | 1.452 | 0.046 | 0.637 | Lipids and lipid-like molecules |
| Theobromine | POS | 94.22 | 181.072 | 18.223 | 1.188 | 1.976 | 0.039 | 15.334 | Organoheterocyclic compounds |
| 1-Methylguanine | POS | 166.41 | 166.072 | 0.680 | 1.904 | 1.719 | 0.044 | 0.357 | Organoheterocyclic compounds |
| Palmitoylethanolamide | POS | 38.47 | 300.289 | 21.279 | 57.782 | 1.667 | 0.025 | 0.368 | Organic acids and derivatives |
| LysoPE(0:0/14:0) | POS | 226.64 | 426.261 | 4.493 | 17.964 | 1.871 | 0.038 | 0.250 | Lipids and lipid-like molecules |
